# Treatment of T3 laryngeal cancer in the Netherlands: a national survey

**DOI:** 10.1186/s13014-015-0440-6

**Published:** 2015-06-26

**Authors:** Patricia Doornaert, Chris H. J. Terhaard, Johannes H. Kaanders, On behalf of the Dutch National Platform Radiotherapy Head and Neck Cancer

**Affiliations:** Department of Radiation Oncology, VU University Medical Center, de Boelelaan 1117, 1081 HV Amsterdam, The Netherlands; Department of Radiation Oncology, University Medical Centre Utrecht, Utrecht, The Netherlands; Department of Radiation Oncology, Radboud University Nijmegen Medical Centre, Nijmegen, The Netherlands

**Keywords:** Larynx cancer, T3 larynx cancer, National survey, Radiotherapy

## Abstract

**Background:**

Treatment strategies for T3 laryngeal carcinoma include radiotherapy (RT) with or without chemotherapy (CT) and sometimes surgery. We conducted a national survey to determine how T3 laryngeal carcinoma is currently being managed in the Netherlands.

**Methods:**

A questionnaire on general treatment policy, also inquiring details on RT and CT, was sent to all 13 radiotherapy departments accredited for treatment of head and neck cancer (HNC) in the Netherlands.

**Results:**

Twelve centers completed the questionnaire. All centers reported using RT with or without CT. Upfront laryngectomy is rarely performed.

At 9/12 centers, CT is added to RT in cases with large tumors in T3N0 disease. Three centers use a volume criterion (3–6 cc); 6 centers don’t specify “large” with such criteria. CT consists of cisplatin 3-weekly (7 centers) or weekly (2 centers), unless contra-indicated or age; 6 centers use an age limit of 70 years. RT is given concomitantly with CT 5×/week except at the 2 centers where cisplatin weekly is combined with 6 fractions/week. In case of RT only, treatment is accelerated. Lymph node levels II-IV are treated electively.

In T3N+ disease, 11/12 centers treat non-bulky T3N1 with RT only. Volume criteria for combined CT-RT are the same as above. Two centers perform an upfront neck dissection in case of (resectable) N3 disease; 10 centers treat T3N2-3 cancer with primary CT-RT, 2 centers don’t use the N-stage criterion.

Total RT dose is 68–70 Gy, the elective dose varies between 46 and 57.75 Gy. Eight centers use a simultaneous integrated boost technique.

**Conclusions:**

Treatment of T3 laryngeal cancer in the Netherlands is generally comparable, with CT-RT for voluminous T3N0 and most T3N+ tumors, but there are some differences between the centers in the use of chemotherapy and the dose-fractionation schemes. Therefore, the aim of the National Platform RT HNC is further standardization of RT dose, fractionation and delivery techniques.

## Background

Laryngeal cancer is relatively rare, with an estimated incidence of 12,630 new cases in 2014 out of a total number of 1,665,540 new cancer cases in the United States [1]. In the Netherlands, the yearly incidence of laryngeal carcinoma is approximately 700 patients out of a total of around 100,000 new cases of cancer [http://www.cijfersoverkanker.nl/selecties/Incidentie_long_en_luchtpijpkanker/img556836474c735].

Almost all laryngeal malignancies occur in the glottis (2/3) and supraglottis (1/3), with subglottic tumors accounting for only 2 % [2]. More than 80 % of glottic tumors are diagnosed at an early stage, whereas in supraglottic cancer this is around 30 % [3]. These tumors are treated with single modality therapy, i.e. RT or transoral (laser) surgery, with 5-year survival rates above 85 % for stage I and around 75 % for stage II [4, 5].

In the 1990s, after the Veterans Affairs laryngeal preservation study showed comparable survival outcomes between total laryngectomy and induction CT followed by RT [6], treatment of stage III–IV locoregionally advanced laryngeal cancer shifted away from surgery towards primary organ preservation strategies. A large retrospective survey on treatment of T3 larynx cancer in the Netherlands demonstrated an inferior outcome with split course RT compared to definitive RT, upfront surgery or RT followed by surgery, leading to abandonment of the split course RT strategy [7]. A decade later, RTOG 91–11 showed concurrent chemoradiotherapy (CT-RT) to be superior to induction CT followed by conventionally fractionated RT or conventionally fractionated RT alone for locoregional control (78 % versus 61 and 56 %, respectively at 2 years) in stage III–IV laryngeal cancer [8]. Around the same time, studies with accelerated radiotherapy (AR) also reported local control rates of up to 80 % [9–12].

Early stage larynx cancer is treated with radiotherapy alone, whereas for T4 tumors total laryngectomy or chemoradiotherapy is the treatment of choice. In T3 laryngeal cancer however, guidelines are less clear. National comprehensive cancer network (NCCN) guidelines advocate CT-RT, single modality RT (in patients unfit for or refusing chemotherapy), laryngectomy (if organ preservation is deemed impossible) or induction CT followed by RT with or without concomitant CT (preferably within a trial) [NCCN guidelines 2–2014]. The 2010 Dutch guidelines recommend organ sparing treatment (if possible) by using concomitant CT-RT or accelerated RT; no volume threshold is stated [http://www.oncoline.nl/index.php?pagina=/richtlijn/item/pagina.php&richtlijn_id=666].

A uniform approach to the treatment of T3 laryngeal cancer would be desirable in order to achieve the best outcome in terms of tumor control and toxicity. However, the relatively low incidence, combined with the fact that (1) these patients are managed in multiple centers and (2) several treatment options are used in the current national guidelines could hamper this uniformity. We therefore conducted a survey to identify how T3 laryngeal carcinoma is currently being managed in the Netherlands. For more than 2.5 decades, treatment of HNC in the Netherlands has been centralized and restricted to the centers of the Dutch Head and Neck Oncology Cooperative Group (DHNOCG, Nederlandse Werkgroep Hoofd-Halstumoren) and their affiliated centers. A questionnaire was sent to the radiotherapy departments of the 8 centers that together make up the DHNOCG and the 5 preferred partner centers. These latter centers have committed themselves to use the same treatment protocols as the related DHNOCG center.

## Methods

The centers of the DHNOCG and the preferred partners were invited to participate in the survey. In addition to asking about the general approach to treatment, we also collected detailed information about the practice of RT and CT-RT at the various centers.

The questions were emailed to the radiation oncologists of these 13 centers who also are the members of the National Platform RT HNC (Landelijk Platform Radiotherapie Hoofdhals Tumoren), an assembly of radiation oncologists representing the 8 DHNOCG centers and their affiliated centers.

The survey included the following questions:

- How is a T3N0 laryngeal carcinoma treated at your center?
- How is a T3N+ laryngeal carcinoma treated at your center?
- Is there a difference in treatment between a supraglottic and glottic tumor?
- Which elective lymph node levels are treated?
- Does tumor volume play a role in your treatment selection?
- Does nodal stage or volume play a role in your treatment selection?
- What is your preferred choice of chemotherapy or biological therapy and do you have an upper age limit for these therapies?

An additional questionnaire was included with specific questions on the radiotherapy treatment; these questions are listed on Table 1.

**Table 1:** Questionnaire on radiotherapy treatment

Preparation
Do you perform an MRI in a mask?
Is your diagnostic MRI matched with the planning CT for delineation?
Do you obtain a PET-CT in mask?
Is your diagnostic PET-CT matched with the planning CT for delineation?
Delineation-Margins
What is your gross tumor volume (GTV)-clinical target volume (CTV) margin (boost volume)?
What is your GTV-CTV margin (elective volume)?
What is your planning target volume (PTV) margin (boost volume)?
What is your PTV margin (elective volume)?
Dose- Technique
What is your total boost dose?
What is the fraction size for the boost dose?
What is your total elective dose?
What is the fraction size for the elective dose?
Do you use a simultaneous integrated boost (SIB) or sequential boost (SEQ?
Do you use static intensity modulated radiotherapy (IMRT) or volumetric modulated arc therapy (VMAT)?
What is your protocol for position verification and correction during treatment?

The results were collected in the spring of 2014, with an update and additional questions in December 2014 and a final update in May 2015.

## Results

All 8 DHNOCG centers and 4 preferred partners completed the questionnaire. The responses are summarized below.

### T3N0 larynx carcinoma

All centers use primary RT, with or without concurrent CT. Five centers report the option of upfront total laryngectomy in exceptional cases (i.e. stridor or a non-functional larynx). At 9 centers the decision to combine RT with CT is based on tumor volume; 3 centers did not report a primary tumor volume criterion.

### T3N+ larynx carcinoma

Except for “non-bulky” T3N1 laryngeal tumors, almost all T3N+ cancers at the centers are treated with concomitant chemoradiotherapy. Two centers do not use an N-stage criterion.

Two centers reported that patients presenting with a non-fixed N3 lymph node undergo an upfront neck dissection, followed by CTRT for the primary tumor and elective neck dissection. Two other centers recently abandoned the strategy of upfront neck dissection.

### Chemotherapy indication and regimens

The indications for combining chemotherapy with radiotherapy were reported as follows:

- volume of the primary tumor: cut-off volume of 3.5 cc (2 centers) or 6 cc (1center) for glottic tumors, cut-off volume of 6 cc for supraglottic tumors: 3 centers, “bulky” tumor: 6 centers
- nodal stage: N1: 1 center, N2a: 10 centers, N2b: 9 centers, N3: 10 centers (of which 2 centers sometimes perform an upfront neck dissection)
- suspicion of extranodal spread on imaging: 4 centers
- submucosal spread: 1 center
- subglottic extension: 1 center

The final decision to give chemotherapy is almost always based on a combination of these factors.

Ten centers reported that their treatment regimen of choice is cisplatin 100 mg/m^2^ 3 weekly and 2 centers reported 40 mg/m^2^ weekly. Once center uses cisplatin for T3N0, but carboplatin-5FU in case of T3N+ carcinoma. If patients are deemed unfit to tolerate the 3-week regimen, cisplatin 40 mg/m^2^ weekly (3 centers) or 6 mg/m^2^ daily (1 center) is given. In case of a contra-indication to cisplatin treatment (e.g. impaired renal function, vascular morbidity) or a World Health Organization (WHO) performance score >1, cetuximab is considered by 6 centers.

Above the age of 70 years, 3 centers reported that they usually combine radiotherapy with cetuximab when the patient is very fit. The other centers did not report combined therapy for this age group.

### Elective lymph node level treatment

For both glottic and supraglottic tumors, all centers treat bilateral lymph node levels II–IV electively. For subglottic tumors or subglottic extension, level VI is included. In case of nodal involvement, level V is included. When level IVa is affected, level IVb is included. All centers use the consensus guidelines published by Gregoire et al. [13].

### Radiation treatment preparation

For T3N0 laryngeal cancer, 1 center uses CT scanning for diagnostic workup; at 11 centers a diagnostic MRI scan is made. This MRI scan is fused with the planning CT scan for contouring purposes. In 3 centers, the diagnostic MRI scan is made in treatment position (i.e. in the treatment mask): in 2 centers routinely, in 1 center only for selected cases. In 1 other center, a second MRI scan is performed in treatment position. In 2 centers, a PET-CT in treatment position is made for both diagnostic and contouring purposes.

For T3N+ laryngeal cancer, all centers use (diagnostic) MRI scans for contouring. Seven centers perform a PET-CT scan in treatment position, which is then used for both diagnostic and delineation purposes.

### Margins

The margin from the GTV to the CTV_boost_ is 5 mm (8 centers), 6 mm (1 center) or 10 mm (2 centers). One center reported a margin of 10 mm for the primary tumor and 5 mm for the involved lymph nodes. To construct an elective margin around the GTV, 5 mm (2 centers), 10 mm (8 centers) and 15 mm (2 centers) were reported. Margins from CTV to PTV are 3 mm (2 centers), 4 mm (2 centers) and 5 mm (8 centers). Some centers use an additional margin in cranio-caudal direction for laryngeal movement.

### Fractionation, dose and technique

When RT alone is used, all 12 centers reported accelerated treatment. At 6 centers an upper age limit of 70 years for AR was reported. Ten centers use 6 fractions per week (i.e. DAHANCA scheme); 2 centers apply an ‘accelerated scheme only’ (ASO-scheme: weeks 1–2: 2 Gy/day on boost and elective volume, 5 days/week (total 20 Gy), week 3–5: 1.5 Gy on boost volume, 5×/week (morning) and 1.8 Gy on boost and elective volume, 5×/week (afternoon) (total 69.5 Gy on boost volume, 47 Gy on elective volume) [10]. When combined with chemotherapy, 10 centers stated that radiotherapy is conventionally fractionated (i.e. 1 fraction/day), while 2 centers combine weekly cisplatin with 6 fractions/week (i.e. DAHANCA scheme).

All centers use a 2-level dose prescription (i.e. for PTV_boost_ and PTV_elective_), without intermediate dose. Fractionation schemes are somewhat diverse (Table 2). Dose prescription is set to 95 % of the dose covering at least 95 % of the boost PTV at 2 centers, 98 % at 8 centers and 99 % at 2 centers, while keeping the volume receiving >107 % as small as possible.

**Table 2:** Fractionation schemes

	SIB	SIB	SIB	SIB	SIB	ASO	SEQ
Boost dose: fraction	2	2	2	2	2	1.5/1.8/2	2
Boost dose: total	68	68	68	70	70	69.5	70
Elective dose: fraction	1.48	1.5	1.55	1.55	1.65	1.8/2	2
Elective dose: total	50.3	51	52.7	54.25	57.75	47.5	46

*SIB* simultaneous integrated boost scheme, *ASO* accelerated scheme only, *SEQ* sequential scheme

Eight centers reported that they always use a pure simultaneous integrated boost (SIB); 6 of these centers have a single scheme, while the 2 remaining centers use 2 different schemes, depending on whether or not chemotherapy is added. The total boost dose varies between 68 and 70 Gy (2 Gy fraction dose); the total elective dose varies between 50.3 and 57.75 Gy with fraction doses varying between 1.48 and 1.65 Gy.

In case of radiotherapy only, 2 centers use the ASO scheme, which is a combination of a sequential and an integrated approach [10]. When concomitant CT-RT is given, these centers use a SIB or sequential scheme. The 2 remaining centers mostly use a sequential scheme, with a fraction dose of 2 Gy and a total dose of 46 Gy to the PTV_elective_ and 70 Gy to the PTV_boost._ Sometimes a SIB scheme is given, i.e. in cases of N+ disease near the parotid glands. The PTV_boost_ then receives 70 Gy (2 Gy/fraction), the PTV_elective_ 54.25 Gy (1.55 Gy/fraction).

Six centers use VMAT, the 6 other centers use static IMRT.

### Set-up protocol and position verification (Image Guided Radiotherapy (IGRT))

Three centers perform daily kilovoltage (kV) planar imaging with online corrections. Additional cone beam CTs (CBCTs) are used to monitor anatomical/soft tissue changes: at 1 center, weekly CBCT scans are made, 1 center makes CBCT scans in the first and fourth week (or weekly if volume changes are anticipated); the remaining center performs CBCT scans only in case of large clinical changes.

Seven centers use only CBCT scans (with offline review) for set-up. Five centers use a no action level (NAL) protocol with a scan made on days 1–3 and a control scan (after the resulting shift has been carried out) on day 4, and thereafter weekly CBCT scans (and correction and a new NAL series if necessary). One center performs 3 CBCT scans in the first week and weekly scans in the following weeks. One center uses daily CBCT set-up with an offline correction protocol.

Two centers use megavoltage (MV) planar imaging with a NAL correction protocol and perform CBCT scans on indication (i.e. when relevant anatomical changes are expected).

## Discussion

The results of this survey, investigating current practice in the Netherlands for T3 laryngeal cancer, show that the majority of centers take a rather similar general approach to T3N0 and T3N+ disease, but the details of the RT or CT-RT approaches differ. All centers use primary radiotherapy for the primary tumor, with or without concomitant chemotherapy/biological therapy, and with or without upfront neck dissection when deemed necessary. Upfront total laryngectomy is performed rarely.

In T3N0 cancer, 9/12 centers use volume criteria to choose between RT alone or concomitant CT-RT. Three centers specify a volume, 6 centers use “bulky” as a criterion and 3 centers don’t use a primary tumor volume criterion. The impact of laryngeal tumor volume on tumor control after RT alone has been investigated in several studies [14, 15]. Cut-off values of 3.5 and 6 cc were reported for glottic and supraglottic cancer respectively [16, 17]. However, Van Bockel et al. also found significant associations with outcome using tumor volume as a continuous variable [15]. In their analysis, tumor volume was a better predictor for disease free survival than T-stage, leading to their suggestion to incorporate tumor volume in T-staging, as it is done in oro- and hypopharyngeal cancer. This was not confirmed by Janssens et al.; in the large Dutch phase III ARCON trial, neither primary tumor volume, nor total nodal volume was a prognostic factor [18]. Interestingly, the 3 centers that did not report a primary tumor volume criterion also took part in the ARCON trial. However, it is still not convincingly clear if cut-off volumes should be used, and if so, which ones.

The main justification for combining chemotherapy with radiotherapy is to increase local control, hopefully leading to a higher rate of larynx preservation. In the RTOG 91–11 study, at a follow up of 3.8 years, the larynx preservation rate was significantly higher in the concomitant CT-RT arm than the induction CT arm and RT alone arm (84 % versus 72 and 67 %) [8]. A recent retrospective series by Al Mamgani et al. showed significantly improved local control (89.6 % versus 68 %) and laryngectomy-free survival (76.8 % versus 53.5 %) at 3 years in favor of CT-RT versus RT alone for T3 larynx cancer, with both groups receiving 6 fractions a week [19]. Conversely, several groups have also reported favorable local control with altered fractionation radiotherapy alone, although these studies were not limited to (T3) larynx cancer. DAHANCA 6 and 7 had 70 % overall 5-year locoregional control with AR [9]. Terhaard et al. reported a 3-year local control rate of 78 % for locally advanced laryngeal cancer treated with the ASO scheme [10]. A phase II study combining AR with carbogen and nicotinamide (ARCON) in 215 HNC patients showed an actuarial 3-year local control rate of 80 % for T2-4 larynx cancer [11]. A subsequent phase III trial in 345 patients resulted in a local tumor control rate at 5 years of 78 % for AR versus 79 % for ARCON, with larynx preservation rates of 84 and 87 %, respectively [12]. Notably, in this large trial no differences in local control between T-stages were seen.

Because patients were enrolled in RTOG 91–11 between 1992 and 2000, staging was done according to the 4^th^ and 5^th^ edition of the American Joint Commission on Cancer. However, paraglottic space invasion and minor thyroid cartilage erosion were added to the staging system only in the 6^th^ edition (2003–2009). At that time, some cases that are currently staged as T3 laryngeal carcinomas would have been classified as a T2 tumor and treated accordingly. The superiority of concomitant CT-RT treatment, as a conclusion of the RTOG 91–11 study, may therefore not apply to all T3 laryngeal carcinomas. This is reflected in the Dutch guidelines, suggesting either primary radiotherapy or CT-RT for T3 laryngeal carcinomas [http://www.oncoline.nl/index.php?pagina=/richtlijn/item/pagina.php&richtlijn_id=666].

In the present survey, for T3N+ laryngeal cancer, nodal stage is considered more important than primary tumor volume for the addition of chemotherapy. One center reported using CT-RT in all cases. With N2-3 disease, almost all centers combine RT with CT. This is consistent with Vergeer et al., who showed worse regional control in patients with a nodal volume of >14 cc treated with RT alone versus CT-RT [20]. However, the 2 leading centers in the ARCON trial do not use a nodal volume criterion, which is in line with the results of their study [18].

In RTOG 91–11 there was no survival benefit from combined CT-RT. Also, a recent update failed to show an overall survival difference between any of the 3 treatment arms, although there was a trend for less distant metastases in the combined arms [21]. This is probably attributable in part to the possibility of a successful salvage total laryngectomy in case of recurrent disease. Nevertheless, a sub-analysis of the MACH-NC database in laryngeal cancer patients from trials performed before 2000 showed a 4.5 % absolute survival benefit in favor of CT-RT versus RT alone [22].

The downside of combining CT with RT is increased toxicity. In RTOG 91–11, the rate of (acute and late) high-grade toxicity was 82 % for the concomitant CT-RT group versus 61 % for the radiotherapy alone group [8]. In the recently published TREMPLIN trial, comparing CT-RT to cetuximab + RT after induction chemotherapy for larynx preservation, acute toxicity was substantial and dose limiting, with only 42 % of patients in the CT-RT arm receiving all 3 cycles of chemotherapy [23].

In this survey, the most important argument raised by the centers in favor of chemotherapy in less advanced cases is that the expected reduced risk of recurrent disease outweighs the toxicity of a combined treatment. Centers that are more reluctant to use chemotherapy (i.e. centers that took part in the ARCON study and their affiliated centers), argue that 5-year local control rates in this large study were high, also in the accelerated-only arm, and that neither tumor volume nor nodal volume was a prognostic factor [12, 18].

Considering the RT schemes, there are minor variations in total boost dose, varying between 68 and 70 Gy. The 2 centers in the present study that reported using 68 Gy were also the leading centers in the ARCON trial, in which the boost dose was set to 68 Gy (in the accelerated-only arm). The dose to the elective volume differs more, with a total dose between 50.3 and 57.75 Gy in the SIB schemes, a dose of 46 Gy in the sequential scheme, and a dose of 47.5 Gy in the ASO scheme. The reason for a higher dose in the SIB schemes is that the total treatment time of the elective PTV is prolonged from 4–5 weeks (in the sequential and ASO scheme) to 6–7 weeks. Although no reliable data are available on the effect of prolonging overall treatment time on control (or loss of control) in elective nodal regions, it is believed it should be compensated for. Since no robust data exist, the elective doses or fractions vary between the centers. Despite these differences, reported Dutch regional outcomes are good to excellent [10–12, 19].

Accurate and precise contouring of the tumor using MRI in treatment position could possibly lead to smaller CTV margins. All centers reported performing a diagnostic MRI for T3N+ larynx cancer, but this scan is made in treatment position at only 4 centers. Although not yet in practice at all centers, most centers in the Netherlands are working on MRI and PET-CT imaging in the treatment mask.

There are some differences in IGRT practice. Centers with Varian equipment (Palo Alto, Ca, USA) perform daily online kV imaging, combined with periodical CBCT imaging, while users of Elekta (Stockholm, Sweden) primarily do CBCT imaging with offline correction protocols. At all but 4 centers, both approaches are combined with PTV margins of no less than 5 mm, although Van Asselen et al. showed that the mean parotid dose increased with increasing margin by approximately 1.3 Gy per mm [24]. Co-registration of MRI imaging in treatment position, combined with well considered set-up protocols, could possibly lead to smaller treatment volumes.

With the increased use of non-surgical treatment for larynx cancer, it is relevant to look at trends in survival. Chen et al. retrospectively reviewed more than 19,000 patients treated between 1996 and 2002 for advanced stage laryngeal cancer and found that treatment with a non-surgical procedure was associated with a higher risk of death. This risk was reduced when treatment was performed at a high-volume center, in which case survival was better compared to a low-volume center [25]. A recent report based on 21 RTOG trials showed the same, with better overall survival in high-accruing centers [26]. This indicates the need for centralization of treatment of low-incidence/high-impact cancer like T3 laryngeal carcinoma. In the Netherlands, this is well organized through the Dutch Head and Neck Oncology Cooperative Group, established 30 years ago to improve knowledge and cooperation, and to centralize treatment of head and neck cancer in the Netherlands. Nevertheless, despite centralization of treatment, some differences between the centers continue to exist in interpreting and implementing the guidelines and the available literature, which is sometimes conflicting. Therefore the current aim of the National Platform RT HNC is to standardize the RT dose and fractionation. The results of this survey have been discussed in the platform and this has already led to some local practice changes. Certain technical issues will also be harmonized, such as planning and imaging protocols. In the future, we hope to collect and analyze outcome data of treatment of larynx cancer in the Netherlands; these results could help to further harmonize treatment protocols.

## Conclusions

As reflected by this survey, treatment strategies of T3 laryngeal cancer in the Netherlands are rather comparable between the 8 DHNOCG centers and their affiliated locations, but differences exist, especially when it comes to the addition of concomitant chemotherapy. Radiotherapy alone is reserved for small volume T3N0 and T3N1 tumors, but not all centers use CTRT for higher N-stages. Since there are also some discrepancies in the details of the RT treatment between centers, the aim of the National Platform RT HNC is to is further standardize the RT dose, fractionation and delivery techniques.

## Abbreviations

AR: Accelerated radiotherapy
ASO: Accelerated scheme only
CBCT: Cone beam CTscan
CT: Chemotherapy
CT-RT: Chemoradiotherapy
CTV: Clinical target volume
DHNOCG: Dutch Head and Neck Oncology Cooperative Group
GTV: Gross tumor volume
HNC: Head and neck cancer
IMRT: Intensity modulated radiotherapy
kV: Kilovoltage
MV: Megavoltage
NAL: No action level
NCCN: National Comprehensive Cancer Network
PTV: Planning target volume
RT: Radiotherapy
SEQ: Sequential scheme
SIB: Simultaneous integrated boost
VMAT: Volumetric modulated arc therapy
